# The Validity and Reliability of a Tire Pressure-Based Power Meter for Indoor Cycling

**DOI:** 10.3390/s21186117

**Published:** 2021-09-12

**Authors:** Nicholas J. Fiolo, Hai-Ying Lu, Chia-Hsiang Chen, Philip X. Fuchs, Wei-Han Chen, Tzyy-Yuang Shiang

**Affiliations:** 1 Department of Athletic Performance, National Taiwan Normal University, Taipei 106, Taiwan; nicholas.j.fiolo@ntnu.edu.tw (N.J.F.); philip.fuchs@ntnu.edu.tw (P.X.F.); gn01800083@gmail.com (W.-H.C.); 2 Office of Physical Education, National Pingtung University of Science and Technology, Pingtung 912, Taiwan; mno2jrter@gmail.com (H.-Y.L.); doof75125@mail.npust.edu.tw (C.-H.C.)

**Keywords:** SRM, cycling technology, validation, tire pressor sensor

## Abstract

The purpose of this study was to evaluate the validity and reliability of a tire pressure sensor (TPS) cycling power meter against a gold standard (SRM) during indoor cycling. Twelve recreationally active participants completed eight trials of 90 s of cycling at different pedaling and gearing combinations on an indoor hybrid roller. Power output (PO) was simultaneously calculated via TPS and SRM. The analysis compared the paired 1 s PO and 1 min average PO per trial between devices. Agreement was assessed by correlation, linear regression, inferential statistics, effect size, and Bland–Altman LoA. Reliability was assessed by ICC and CV comparison. TPS showed near-perfect correlation with SRM in 1 s (*r*_s_ = 0.97, *p* < 0.001) and 1-min data (*r*_s_ = 0.99, *p* < 0.001). Differences in paired 1 s data were statistically significant (*p* = 0.04), but of a trivial magnitude (*d* = 0.05). There was no significant main effect for device (F(1,9) = 0.05, *p* = 0.83, $\eta_{p}^{2}$ = 0.97) in 1 min data and no statistical differences between devices by trial in post hoc analysis (*p* < 0.01–0.98; *d* < 0.01–0.93). Bias and LoA were −0.21 ± 16.77 W for the 1 min data. Mean TPS bias ranged from 3.37% to 7.81% of the measured SRM mean PO per trial. Linear regression SEE was 7.55 W for 1 min TPS prediction of SRM. ICC_3,1_ across trials was 0.96. No statistical difference (*p* = 0.09–0.11) in TPS CV (3.6–5.0%) and SRM CV (4.3–4.7%). The TPS is a valid and reliable power meter for estimating average indoor PO for time periods equal to or greater than 1 min and may have acceptable sensitivity to detect changes under less stringent criteria (±5%).

## 1. Introduction

Accurate and systematic quantification of exercise intensity is an integral component to monitor and prescribe exercise [1] for both athletic [2] and health-related goals [3]. Exercise intensity can be broadly dichotomized into internal, relating to an individual’s specific acute stress response (e.g., heart rate), and external, independent of the individual’s response (e.g., average running speed) [1]. Ideally, the individual relationship between internal and external intensity can be made to allow for a more complete evaluation of the dose of exercise and the consequential responses of physiological and performance changes. External intensity in cycling can be quantified as power output (PO) and measured with a cycling-specific power meter.

Cycling PO is the mechanical work rate (Watts, W) during cycling, expressed as the torque applied to the pedals and the pedaling rate (revolutions per minute, rpm). Unlike heart rate, which can lag effort, or speed, which is influenced by terrain and wind, PO is an objective way to assess the instantaneous cycling intensity. Further, PO can be linked to the physiological- (e.g., VO2max) [4] or performance-based (e.g., critical power) [5] characteristics of the individual for more guided exercise prescription. Thus, access to PO may be of value to both athletic and health-minded cyclists.

Specific cycling power meters are required for the quantification of PO. A variety of technologies exists to integrate a power meter onto a bicycle, such as crank-based [6,7], pedal-based [7,8,9], bottom bracket-based [10], and hub-based [7,11] power meters. The crank-based SRM (Schoberer Rad Messtechnik, Juelich, Germany) power meter has been validated [6,12] and is considered the “gold standard” reference in evaluating other bicycle-integrated power meters [7,8,9,10,11,13]. Additionally, the SRM was deemed an appropriate tool for detecting meaningful changes in performance in trained cyclists, due to its high degree of accuracy and reliability [14]. The accuracy of the SRM was reported at <1–3% during steady and intermittent cycling when compared to a dynamic cycling rig [12].

Most bicycle integrated power meters, including the SRM, use strain gauges to measure torque as part of the power calculation methodology. These strain gauges are commonly located in the pedals, bicycle crank (crank arm or gear ring spider), or rear wheel hub. Most commercially available strain gauge power meters, such as the PowerTap hub-based power meter [7,11], Assioma Favero pedal-based [9], and Powertap P1 pedal-based [8,13] have shown strong correlation and acceptable agreement with the SRM, and produce reliable data. However, some inconsistencies and questionable validity and reliability have also been reported for strain gauge-based power meters. Hutchinson and colleagues [15] reported greater disagreement between the Garmin Vector pedal-based power meter and the SRM than those reported in other investigations [7,16,17]. Mixed findings were also reported for the crank arm-based Stages power meter. A large disagreement (25 W bias) between the Stages and the SRM during outdoor off-road cycling [18], whereas greater agreement was reported for indoor cycling [7,19]. The Stages were deemed acceptable for road cycling when more stringent (<3%) error margins are not needed [19]. Poor reliability results were reported for the Look Keo pedal-based system when compared against the SRM [20].

Although the use of strain gauges is common, other methodologies of power calculation have been utilized for bicycle-integrated power meters. However, the reported accuracy of these devices tends to show weaker agreement with the SRM, particularly when compared to the hub-based strain gauge power meters. For example, the Polar S710 power meter calculates power from bicycle chain speed and tension variables derived from chain vibrations [21]. The average power output over 5–6 min of constant cadence cycling was compared between the S710 and the SRM [21]. Although the S710 displayed a strong correlation (r > 0.99) with the SRM, the S710 tended to overread the average power during constant cadence cycling tended to by approximately 4% and 7% for indoor and outdoor cycling, respectively. These findings lead the authors to conclude that this device was acceptable for recreational cyclist use when accuracy criteria are less stringent. A second non-strain gauge methodology reported in the literature is the use of optoelectronic sensors to evaluate torsion at the bottom bracket to estimate cycling power [10]. However, the accuracy and reliability of this methodology were reported to be less favorable than a hub-based strain gauge power meter, when evaluated against the SRM [10]. Power readings were 6.3% higher and 12.0% higher during indoor and outdoor cycling with this methodology over the SRM [10].

Although current commercially available power meters are generally valid and reliable tools, practical limitations to widespread adoption exist. First, these devices may be cost-prohibitive. Second, the hardware dependence (cranks, pedals, hubs, etc.) restricts ease of integration into a variety of different bike types. For example, crank-based and hub-based power meters require changing a major component of the bicycle and pedal-based meters require the use of specialized cycling shoes and cleats. Therefore, the use of power meters is largely limited to professional and serious amateur road cyclists. Given the potential health benefits of cycling as a leisure activity [22] and the importance of intensity in exercise prescription [3], making power-based cycling accessible to the larger population may have positive and substantial public health outcomes.

**Figure 1:**
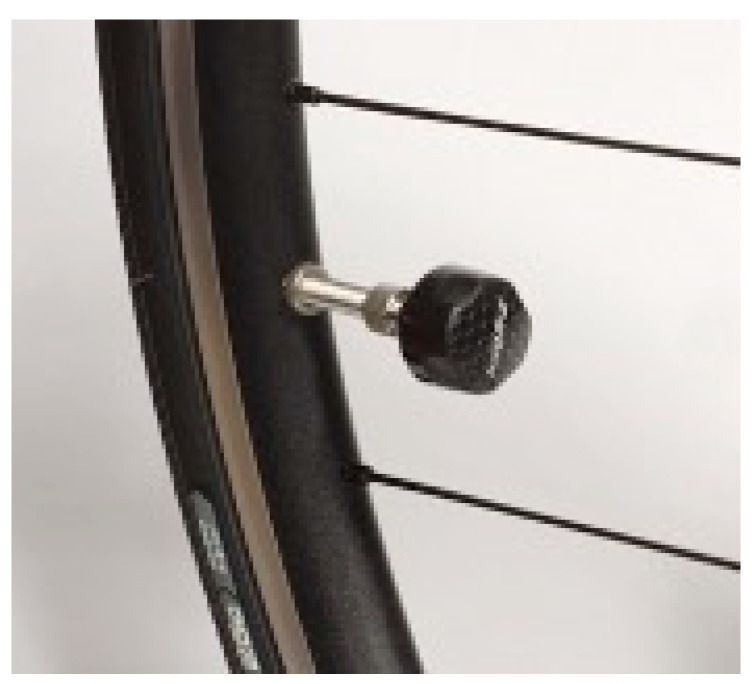
The tire pressure sensor is attached to the valve stem of the bicycle rear wheel.

In contrast to the use of strain gauges such as direct-measurement power meters, a recently developed, commercially available tire pressure sensor (TPS) power meter estimates PO through fluctuations in tire pressure (Figure 1) and pedaling rate. The tire pressure sensor is attached to the valve stem of the bicycle rear tire and sends real-time data to a cycling computer via a Bluetooth connection. This methodology is not bound to the before-mentioned practical limitations, as it is less expensive than traditional power meters and can be quickly transferred between a variety of bikes. Therefore, if this device is a valid and reliable power meter, it may allow for power-based cycling to become more accessible to the general population. The validity and reliability of the TPS have not previously been evaluated. The purpose of this investigation was to assess the validity and reliability of the TPS against the “gold standard” SRM power meter during indoor cycling.

## 2. Materials and Methods

### 2.1. Participants

This study included 8 male and 4 female recreationally active participants (age: 24.9 ± 3.3 years; mass: 69.83 ± 13.14 kg; height: 169.5 ± 5.9 cm). All participants reported no contraindications to physical exercise. All participants were familiar with cycling, but none were competitive cyclists. Informed consent was obtained from all participants prior to participation. The study was conducted according to the guidelines of the Declaration of Helsinki and approved by the Institutional Ethics Committee of the China Medical University Hospital (approval number: DMR101-IRB1-139).

### 2.2. Design

The validity and reliability of the TPS were compared against the gold standard SRM during steady-state indoor cycling. Participants completed data collection during one laboratory visit. The visit consisted of a familiarization and warm-up session, followed by 8 separate 90 s trials with fixed cadence and chainring-cog gearing (Table 1) for data collection. The familiarization and warm-up consisted of 10 min of cycling at the gearing and rpm condition with the lowest intensity (i.e., “Trial 1”). The trial order was randomly assigned. A metronome was used to assist the participants to keep the appropriate cadence. Real-time cadence was displayed to the participant via a handlebar-mounted bike computer. Participants were given approximately 2 min of rest between trials. Two participants could not keep tempo with the 100 rpm trial and their data from that trial was excluded from overall analyses. In three of the trials, the participant stopped cycling before 90 s or became inconsistent in their pedaling rate. In these cases, the data were manually reviewed to select consecutive data for a sample close to 60 s.

### 2.3. Material

PO was simultaneously collected by a strain gauge power meter (SRM FSA, Schoberer Rad Messtechnik, Juelich, Germany) and a TPS power meter (Arofly X-Elite, Arofly, New Taipei City, Taiwan). A zero offset was performed on the SRM according to the manufacturer’s instructions before every data collection session. The TPS was installed on the bicycle rear wheel as per the manufacturer’s instructions. Tire pressure was standardized to 100 psi prior to TPS installation. PO data for both power meters were simultaneously collected via a Bluetooth-capable bike computer (X-Elite Bike Computer, Arofly, New Taipei City, Taiwan). All PO data were sampled at a frequency of 1 HZ. PO data were downloaded using the manufacturer’s software.

All cycling was performed on an indoor cycling trainer (FG540 Hybrid Roller, Minoura, Gifu, Japan) and on an identical bicycle (TCR Advanced, Giant, Taichung, Taiwan). This hybrid trainer uses dual rollers to create friction on the rear wheel and a stand to keep the front of the bicycle stationary. The input from a magnetic resistance on the rear rollers was disconnected for all trials, allowing for no added resistance. Bicycle seat positioning was standardized to allow for approximately 30–40° of knee flexion and handlebars were adjusted for comfort.

### 2.4. Data Processing and Statistical Analysis

#### 2.4.1. Data Processing

Each 90 s cycling trial was processed to remove the initial and final 15 s of cycling, leaving 60 s of data for analysis. The first and last 15 s were removed to allow the participant to achieve a steady pedaling rate and remove any potential artifacts associating with anticipating the end of the trial. The TPS and SRM PO data were paired by timestamps, allowing for direct comparison of instantaneous PO between devices. RPM data were assessed to ensure each participant complied with the desired cadence.

Data analysis consisted of two segments. Analysis 1 used the instantaneous, 1 s paired data across all participants and trials (*n* = 5612). The intent of this analysis was to describe and classify the general relationship between the two power meters as it relates to the sampling frequency of the TPS (i.e., 1 Hz), as well as provide transparency to the dataset. Analysis 2 compiled the 60 s averaged paired data per participant and trial. Analysis 2 was intended to serve as the main comparison between the two devices because evaluation of isolated, 1 s instantaneous power is not common in practice. One-minute averaged data was chosen because power analysis often involves evaluation of the average power sustained over specific time series greater than 1 min in duration (e.g., 5 min mean maximal power, functional threshold power testing, critical power testing, etc.) [4,5]. Therefore, comparison of 1 min averaged allows for evaluation of the TPS as it may be more commonly used in a real-world application, while also allowing for more transparence to the error that may be reduced with averaging larger timeframes. Data analysis was conducted via Microsoft Excel 365 and IBM SPSS Statistics for Windows, version 26.

Prior to the primary analysis, the Shapiro–Wilk test was conducted to assess data normality and Levene’s test was conducted to assess data for equal variance. Nonparametric tests were performed when data were not normal or lacked equal variance. Descriptive statistics were expressed as the mean ± standard deviation. The median, minimum, and maximum values were presented when nonparametric analysis was performed. Effect sizes were interpreted according to the criteria of Hopkins, Marshall, Batterham, and Hanin [23].

#### 2.4.2. Data Processing

Validity assessment of the 1 s data was completed through a variety of statistical analyses. Spearman’s correlation coefficient (rho, *r*_s_) was used to assess the strength of the relationship between TPS and SRM. The predictive error of the TPS was expressed in absolute units (W) from the standard error of estimate (SEE) and as a percentage (%) from the typical error of estimate (TEE), as calculated through linear regression of the data and log-transformed data, respectively [16,24]. Agreement between devices was assessed with Bland–Altman plots and 95% limits of agreement (LoA = bias ± 1.96∙SD between devices) [25]. The 95% confidence limits (95% CL) for the bias were determined as the bias ± 1.96∙SD between devices/$\sqrt{\mathrm{sample}\;\mathrm{size}}$. The nonparametric Wilcoxon signed-rank test was conducted to assess for statistical differences between the paired PO data by device. Effect size was calculated via gPower 3.1.9.2 (Heinreich-Heine-Universität, Düsseldorf, Germany) as Cohen’s d. Statistical significance was set at *p* < 0.05.

#### 2.4.3. Validity Analysis

Validity assessment of the 1 min trial averages was similar to the 1 s analysis with the following exceptions. A statistical difference between devices was assessed with a two-way repeated-measures ANOVA (trial × device) and partial eta square ($\eta_{p}^{2}$) as effect size. Paired samples *t*-tests were performed for each trial to compare between device means. Cohen’s d was calculated for each comparison. Pearson’s correlation coefficient (*r*) was determined using individual trial TPS vs. SRM data and a complete 1 min data set. Statistical significance was set at *p* < 0.05.

To add greater context to the practical implications of differences between devices, the average TPS bias was expressed as a percentage of the average SRM power per 1 min trial. Practical application was based on different thresholds of 2% [9,14], denoting use for trained athletes needing high sensitivity to small changes, and 5%, denoting use for recreational cyclists needing an acceptable but less stringent sensitivity to small changes. The 5% threshold was chosen because power-based performance reliability can vary greatly as a function of training status [26,27], testing type [14,27,28,29], key performance metric [27,28,29], testing duration [27,30], and relative intensity [31].

#### 2.4.4. Reliability Analysis

The 1 min average PO was normalized by cadence and used for reliability analysis across trials within each gearing level. Normalization by gearing to allow for reliability across all trials was not possible because the mechanical effect of the two gearing levels could not be determined precisely. Two-way mixed, single measurement intraclass correlation coefficients (ICC_3,1_) with 95% confidence intervals (95% CI) and coefficients of variation (CV) across trials were calculated for agreement and variation, respectively, separately for a low and high level of gearing and for each device. Differences in CV between devices were assessed via dependent *t*-tests.

## 3. Results

### 3.1. Validity Analysis 1-S Data

**Figure 2:**
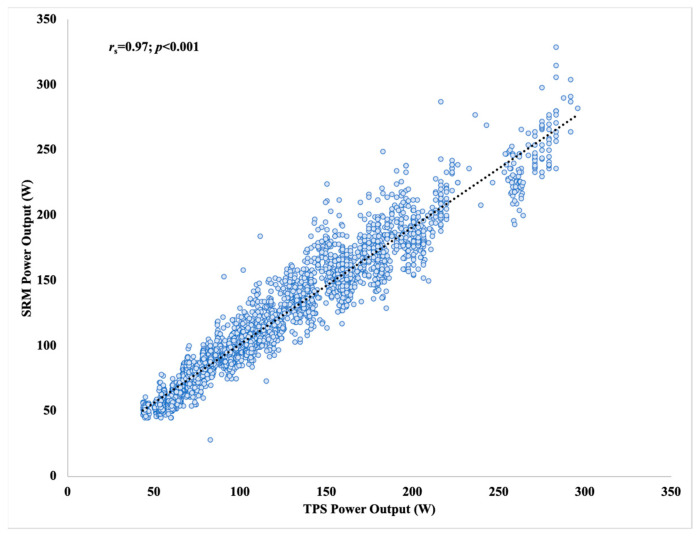
Correlation results of 1 S raw data between devices.

**Figure 3:**
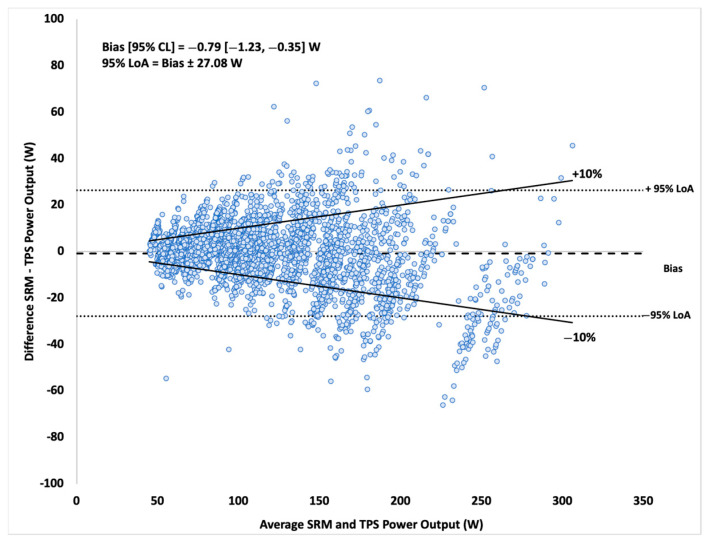
Bland–Altman plot with 95% LoA of 1 S data between devices.

Spearman’s rho correlation coefficient was calculated to evaluate the relationship between the TPS and SRM PO data. Analysis revealed a statistically significant relationship between the two devices, *r*_s_ = 0.97, 95% CL [0.97, 0.97], *p* < 0.001, *n* = 3779. The scatterplot presented correlation results between devices in Figure 2. The Bland–Altman plot displayed bias, 95% CL, and 95% LoA in Figure 3. The Wilcoxon signed-rank test indicated a statistically significant difference (*p* < 0.001) in median PO between TPS (median: 105.84, minimum: 43.63, maximum: 295.85) and SRM (median: 106.01, minimum: 28.00, maximum: 329.00), but magnitude of difference in means (TPS: 115.36 ± 49.53 vs. SRM: 114.13 ± 46.60) was trivial (*d* = 0.02). SEE and TEE of the linear TPS model to predict SRM were 12.83 [12.63, 13.03] W and 10.24 [10.08, 10.41] %, respectively.

### 3.2. Validity Analysis of 1 min Average Trials

**Figure 4:**
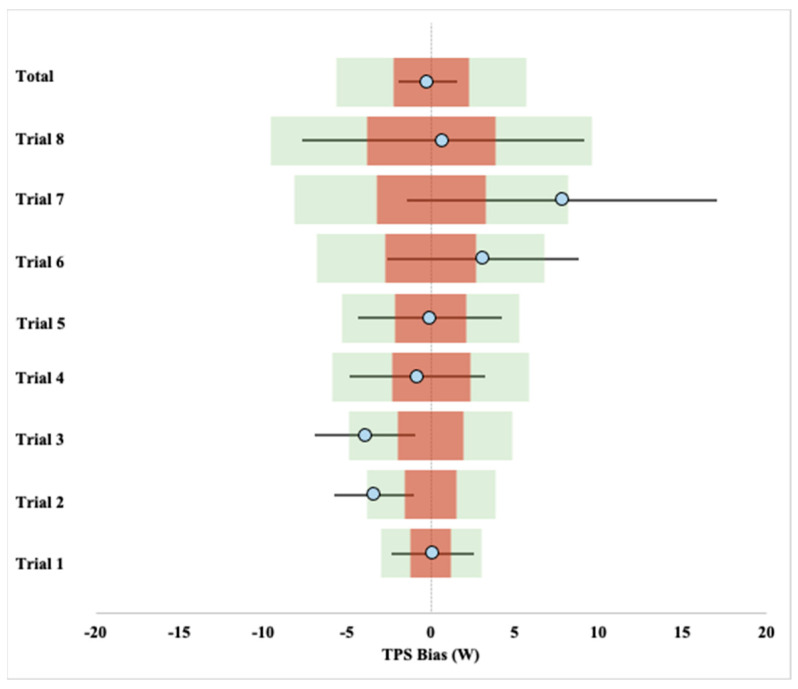
Bias with 95% confidence limits per trial compared with ± 2% and 5% of the SRM mean.

**Figure 5:**
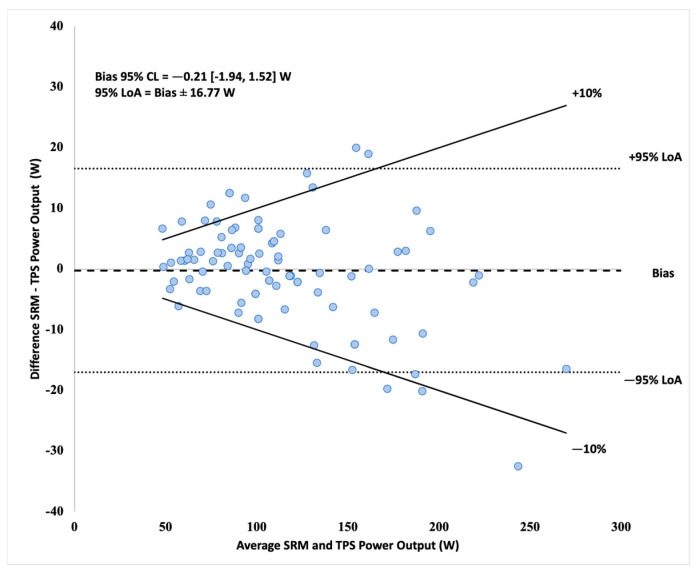
Bland–Altman plot with 95% LoA of 1 min averaged data between devices.

Correlation and linear regression results per trial are presented in Table 2. Bias results were compared against a 2% and 5% tolerance of the mean SRM per trial in Figure 4. The Bland–Altman 95% LoA plot is presented in Figure 5.

The two-way repeated-measures ANOVA showed a significant main effect for trials (F(1.21,10.91) = 250.04, *p* < 0.001, $\eta_{p}^{2}$ = 0.97) but not for devices (F(1,9) = 0.05, *p* = 0.83, $\eta_{p}^{2}$ < 0.01). An interaction between trials and devices was found (F(2.30,20.69) = 5.11, *p* < 0.001, $\eta_{p}^{2}$ = 0.36). Post hoc comparisons between devices for each trial are displayed in Table 3. Statistical differences were found between devices for Trial 2 (TPS: 73.14 ± 14.51; SRM: 76.51 ± 14.49) and Trial 3 only (TPS: 93.68 ± 17.46; SRM: 97.61 ± 17.84). However, the magnitude of difference between devices was moderate and represented an approximate 4% PO difference between devices. No other between-device differences were significant by trial and all other magnitudes of difference were trivial to small.

### 3.3. Reliability of 1 min Average Trials

ICC_3,1_ across trials was 0.96 (95% CI: 0.86–0.99) in the 53 × 15 gearing trials and 0.96 (95% CI: 0.80–0.99) in 53 × 11 gearing in TPS and 0.91 (95% CI: 0.70–0.97) in the 53 × 15 gearing trials and 0.95 (95% CI: 0.87–0.99) in 53 × 11 gearing trials in SRM. CV across trials did not differ between devices in the 53 × 15 gearing trials (TPS: 3.6 ± 1.2%, SRM: 4.7 ± 2.3%; *d* = 0.54, *p* = 0.09) and the 53 × 11 gearing trials (TPS: 5.0 ± 1.5%, SRM: 4.3 ± 1.5%; *d* = 0.50, *p* = 0.11).

## 4. Discussion

The purpose of this investigation was to assess the validity and reliability of a TPS power meter in comparison to the “gold standard” SRM power meter during indoor cycling. The analysis compared both 1 s and 1 min averaged data between devices. Interpretation of the results was primarily based on the 1 min data. In practice, power data is more likely to be used as an average outcome over a given time frame (e.g., 20 min average power), and not as isolated 1 s data. The 1 s data was included to provide greater transparency and to examine the potential limitations of the TPS.

Correlation analysis indicated a near-perfect relationship between the TPS and SRM in the 1 s data (*r*_s_ = 0.97) and 1 min (*r* = 0.99) data sets. This finding is consistent with other validated power meters when compared to the SRM, such as the Powertap (*r* = 0.99 [7,11]), Stages (*r* = 0.99 [7]), Garmin Vector (*r* = 0.97 [16], 0.99 [7]), and Powertap P1 (*r*_s_ = 0.99 [8]). The correlations between devices per individual trials were also near perfect, but of a slightly lower range (*r* = 0.92–0.97). This lower outcome may be influenced by the lower number of data points (*n* = 12) and evaluation over a restricted power range when examined per trial. These correlation results suggest that the TPS can adequately discriminate between greater and lower PO within the tested range (60–250 W).

The ability of the TPS to accurately predict SRM PO was evaluated through SEE and TEE calculation from linear regression models of the 1 s and 1 min data. The 1 s data SEE (12.83 W; 10.24%) and TEE were greater than those for the 1 min (7.55 W; 6.43%). The range of TEE for the individual trials ranged from 4.8% to 8.23%. These 1 min TEE values are similar to, but higher than those reported for 1 min averaged Garmin Vector data (TEE = 2.5 [1.9, 3.5]% [16]; SEE = 4.8–5.4 W [17]. However, the PO during cycling evaluated in this study (approximately 100–400 W) was substantially greater than that in our study (119 ± 48 W), potentially amplifying the effects of small, systematic absolute differences. These results suggest that the TPS may be more appropriately used if evaluating averaged data of 1 min duration or greater duration. An isolated 1 min average is likely to have an error rate within 6.50% of those measured by the SRM within the tested power range (60–250 W).

The magnitude of difference between devices was low, as measured by effect size. The effect size was trivial for both the 1 s (*d* = 0.02) and the 1 min $(\eta_{p}^{2}$ < 0.01) dataset comparisons. The magnitude of differences per individual trial ranged from trivial (*d* = −0.01) to moderate (*d* = −0.93). Noteworthy is that a statistically significant difference between devices existed in the 1 s analysis (*p* ≤ 0.001), but no significant main effect for the devices was present in the 1 min data (*p* = 0.83). The statical finding in the 1 s data may be a function of statistical power achieved with a large sample size (*n* = 5612) [32] and may not represent a practical difference when evaluating PO (i.e., 115 vs. 114 W) as it relates to describing the average work rate over an exercise session. Taken together with the above-mentioned results, these findings suggest that the TPS is likely to provide results of averaged PO similar to the SRM, particularly when large data sets are used. The average bias between the TPS and SRM is similar to those reported in the literature of other power meters against the SRM using 1 min averages. The average bias for the 1 min trials ranged from −3.93 to 7.81 W, representing a relative bias range from approximately −4.0 to 4% deviation from the SRM. The bias between devices was reduced when the 1 s (−1.23 W) and the 1 min (−0.21 W) full datasets were evaluated. Similar bias ranges have been reported for the Powertap P1 pedals (−2.4 ± 4.8 W to −9.0 ± 5.3 W [8]), Powertap hub-based (1.3 ± 6.0 W and 2.9 ± 3.3 W [7,11]), Garmin Vector (0.6 ± 6.2 W [7]), Assioma Favero (−2.7 ± 5.8 W to −6.0 ± 9.9 W [9]) power meters. The TPS performed substantially better in matching the SRM when compared to the ErgomoPro power meter, which yielded an average bias of 14.5 ± 7.7 W [10], and the Stages power meter, which yielded an average bias of −13.7 ± 12.4 W [7]. These findings further suggest that the TPS may provide results similar to the SRM if an average is taken of sufficient sample size. Noteworthy is the drop in bias when results are compared per the individual trial to the overall 1 min dataset. Although a long-duration sample was not collected in this study, the 1 min full data set is a function of 94 separate 1 min averages (94 min of data) and each trial is the function of 12 separate 1 min functions (12 min of data). Although still speculative, if such trends are representative of the averaging effect of long-duration data, these results suggest that the TPS may be highly accurate in providing the average PO over such time durations. Further research is needed to investigate the TPS performance in durations longer than 1 min duration.

Of note is the potential variability of power measurement between devices due to the site of sampling. Conceptually, power meters may give different, yet accurate PO values depending on if the site of measurement is at the crankset or real wheel, due to mechanical inefficiencies along the bicycle chain drive system [7,11]. Mechanical losses from crankset to wheels in PO may be as high as 2–4% [33,34]. However, the TPS did not display consistency in under reading PO, compared to the SRM (Figure 4). Whether or not this inconsistency is due to methodological issues relating to the TPS calculation process itself, or external factors such as cycling technique or temperature changes due to the tire friction on the cycling roller are not known and deserve further investigation.

Even though the measured bias is comparable to other power meters, the relative bias may be too great to be used to monitor small changes in well-trained cyclists (i.e., <2%) [14] Half of the 1 min trials had an average bias outside the 2% tolerance from the measured SRM values, but all were within 5% of the SRM values. Given the variability in power-based performance testing and monitoring [14,29], a tolerance of 5% may be practically acceptable for detecting larger changes in performance (e.g., changes over a season) or for intensity monitoring in recreational cyclists. Therefore, additional testing is needed to further understand the practical tolerance limits of the TPS.

The intra-class correlation coefficient analysis indicated that the TPS behaved consistently and in a manner similar to the SRM across the various gearing and pedalling trials. These results indicate that the TPS is likely not susceptible to reliability concerns arising from changes in the process of cycling (i.e., PO derived from different rpm and gearing configurations). This study used a range of rpm that is common to most recreational cyclists (50–100 rpm). However, gearing was limited to only two configurations (53 × 15 and 53 × 11) and participants did not change gears during the data collection process. Further research is needed to examine the influence of inconsistent pedalling rates and shifting on the reliability of the TPS. Additionally, inter-session reliability of repeated gearing and pedalling configurations were not performed in this study.

There are several limitations to this study that prevent a broader evaluation of the TPS. First, the TPS was not evaluated outdoors. How the TPS will perform in variable terrain and a more ecological setting is yet to be determined. Second, the TPS was only evaluated in the seating position and under a controlled pedalling rate. Standing or highly varied pedalling rates may influence the accuracy of the TPS. Finally, repeated trials of identical gearing and pedalling conditions were not performed. A more complete inter-session comparison is needed to extend the reliability conclusions of the TPS.

## 5. Conclusions

The TPS is a valid and reliable power meter within the testing parameters of this study. The TPS may provide an accurate and reliable assessment of average power over timeframes of 1 min duration or longer on an indoor roller trainer. Enhanced accuracy of the TPS may possibly be achieved if the average PO is reported. Such a tool may be of practical use for general exercise or power testing (e.g., functional threshold power, critical power) for power outputs below 300 W and with less stringent error tolerance (±5%). Further investigation is needed to evaluate the TPS in outdoor use and under additional testing parameters, such as long-duration cycling, standing, sprinting, and inconsistent pedaling.

## Figures and Tables

**Table 1:** Gearing and RPM during each trial.

Trial	Gearing	RPM
1	53 × 15 ^a^	50
2		60
3		75
4		90
5	53 × 11 ^a^	60
6		75
7		90
8		100

^a^ Chain ring tooth × rear cog tooth.

**Table 2:** Correlation and linear regression results by trial 1-minute average power.

Trial	*n*	Correlation	SEE (W) 95% CI [LL, UL]	TEE (%) 95% CI [LL, UL]
Trial 1	12	*r* = 0.96, *p* < 0.001	3.19 [2.23, 5.61]	5.90 [4.11, 10.65]
Trial 2	12	*r* = 0.97, *p* < 0.001	3.90 [2.72, 6.85]	5.48 [3.80, 9.82]
Trial 3	12	*r* = 0.96, *p* < 0.001	4.98 [3.48, 8.73]	5.59 [3.88,10.02]
Trial 4	12	*r* = 0.96, *p* < 0.001	5.87 [4.10, 10.30]	4.90 [3.40, 8.76]
Trial 5	12	*r* = 0.97, *p* < 0.001	4.51 [3.15, 7.92]	4.80 [3.33, 8.58]
Trial 6	12	*r* = 0.95, *p* < 0.001	6.96 [4.86, 12.21]	5.61 [3.89, 10.06]
Trial 7	12	*r* = 0.92, *p* < 0.001	11.92 [8.26, 20.74]	8.23 [5.68, 14.89]
Trial 8	10	*r* = 0.96, *p* < 0.001	10.27 [6.94, 19.68]	6.18 [4.13, 12.17]
Total	94	*r =* 0.99, *p* < 0.001	7.55 [6.60, 8.82)]	6.43 [5.60, 7.56]

**Table 3:** Comparison of trial 1-minute average by device.

	Mean (SD)		
Trial	TPS (W)	SRM (W)	Group Differences
Trial 1	60.74 (12.80)	59.90 (10.96)	*d* = 0.21 ^s^, *p* = 0.48 ^a^
Trial 2	73.14 (14.51)	76.51 (14.49)	*d* = −0.90 ^m^, *p* < 0.01 ^a^
Trial 3	93.68 (17.46)	97.61 (17.84)	*d* = −0.93 ^m^, *p* < 0.05 ^a^
Trial 4	116.91 (22.15)	117.74 (19.92)	*d* = −0.13 ^t^, *p* = 0.66 ^a^
Trial 5	105.61 (21.50)	105.67 (16.91)	*d* = −0.01 ^t^, *p* = 0.98 ^a^
Trial 6	139.01 (27.00)	135.92 (21.96)	*d* = 0.34 ^s^, *p* = 0.26 ^a^
Trial 7	171.73 (36.23)	163.23 (29.22)	*d* = 0.58 ^s^, *p* = 0.07 ^a^
Trial 8	192.28 (40.71)	191.57 (35.32)	*d* = 0.06 ^s^, *p* = 0.86 ^a^
Total	113.32 (48.02)	113.11 (44.55)	$\eta_{p}^{2}$ < 0.01, *p* = 0.83 ^b^

^a^ post hoc planned comparison paired *t*-test. ^b^ main effect from two-way repeated-measures ANOVA. ^t,s,m^ effect size interpretations as trivial, small, and medium effects, respectively [23].
